# Exploring Transient Acute Respiratory Distress in Cholera: A Case Series

**DOI:** 10.1002/ccr3.71356

**Published:** 2025-10-24

**Authors:** Samuel Amo‐Tachie

**Affiliations:** ^1^ University of Ghana Medical School, University of Ghana Accra Ghana; ^2^ Mamprobi Hospital Accra Ghana

**Keywords:** cholera, dehydration, electrolyte imbalance, respiratory distress

## Abstract

Transient acute respiratory distress is a complication of cholera that is not fully understood, even though there are plausible theoretical explanations. Clinicians managing cholera outbreaks need to be aware of this phenomenon in order to manage it appropriately, while further studies are required for a holistic understanding and possible prevention.

## 1 Introduction

Cholera is one of the most lethal infectious acute gastroenteritises of public health importance. It can occur sporadically, endemically, epidemically, and even pandemically, but it is best known for its epidemics, especially in low‐income areas [1, 2].

Cholera is caused by *Vibrio cholerae* , a highly motile, comma‐shaped bacterium [3]. There are several variants, classified according to their unique antigenic properties (serogroups). The most significant are serogroup O1 and serogroup O139, with *V. cholerae* O1 being the most frequent cause of epidemics, as with the one that happened in Ghana [4]. Unlike polio, an eradicable disease that also spreads by fecal‐oral transmission (since it meets all criteria for eradication: being infectious, having humans as primary hosts without long‐term environmental reservoirs, and the availability of vaccines, as well as political and financial support for eradication efforts), cholera is not considered feasibly eradicable because of long‐term environmental reservoirs such as water bodies [5]. The ultimate public health goal is to achieve a near‐eradication state, which is possible since it satisfies the other criteria. Notably, it is one of the diseases in which blood groups appear to play a role in disease severity—the blood group O, which happens to be the commonest in Ghana, is associated with severe disease [6, 7, 8].

There was an outbreak in Ghana by the O1 serotype, which had its peak in the last quarter of 2024. Several efforts, including a mass‐vaccination response, helped abort the outbreak. There was, however, an interesting observed phenomenon that happened in several cases, which was a transient acute respiratory distress (TARD) whose duration ranged from 4 to 72 h. This observation was irrespective of the severity of cholera the patients had.

Below is the description of three such cases in whom this phenomenon was observed in a cholera isolation bay.

## 2 Case Descriptions

### 2.1 Case 1

#### 2.1.1 Case History/Examination

This was a 23‐year‐old male with no underlying medical conditions who presented with diarrhea and vomiting of a day's duration. He had about 10 episodes of each of these. He had moderate dehydration on arrival (increased thirst, rapid pulse, mildly sunken eyes, normal skin turgor) and was afebrile, conscious, and alert. His pulse was rapid, but he had normal blood pressure. There was no form of respiratory distress; his chest was clear to auscultation, and oxygen saturation (SpO_2_) was 98% on room air. Shortly after his morning assessment, he developed an acute shortness of breath. On reassessment, his SpO_2_ was 89% on room air, and his respiratory rate was 30 cycles per minute (cpm), but his chest was still clear to auscultation, and there was no cough or fever.

#### 2.1.2 Differential Diagnosis, Investigations, and Treatment

The differential diagnosis for his shortness of breath included pneumonia, acute pulmonary edema from fluid overload, acute respiratory distress syndrome, and metabolic acidosis with respiratory compensation. Cholera was diagnosed by a rapid diagnostic test (RDT) for cholera by rectal swab, which was confirmed by a stool culture. His complete blood count was unremarkable. A chest x‐ray, unfortunately, could not be done for any of the patients at the isolation bay while they were still having active diarrhea, as there was no mobile x‐ray available, and everyone with respiratory symptoms had resolution by the time the diarrhea had subsided. Again, arterial blood gas could not be done for any of the patients due to the unavailability of blood gas analyzers at the treatment center.

He was resuscitated with Ringer's lactate infusions alongside at least 250 mL of oral rehydration solution (ORS) after each loose stool as he was able to drink. He was supported with intranasal oxygen at 4 L/min, which increased his oxygen saturation to 98%. He received a dose of doxycycline (300 mg) and was monitored for improvement of his shortness of breath.

#### 2.1.3 Outcome

Within 4 h from the onset of his shortness of breath, he felt better again and was weaned off oxygen. His stool frequency decreased significantly to one in 4 h within 6 h of admission, and his pulse normalized. After a total of 24 h on admission, he was discharged, as his vomiting and diarrhea had ceased and was tolerating every kind of meal.

### 2.2 Case 2

#### 2.2.1 Case History/Examination

The second was a 37‐year‐old female, also with no known comorbidities. She had diarrhea and vomiting of a day's duration. She was moderately dehydrated on presentation, as described in the first case. She had no respiratory distress on arrival and had normal SpO_2_ and chest findings. She was passing an average of 8 loose stools per day on admission for the first 2 of her 4‐day admission. On her second day of admission, she was seen to be in sudden‐onset respiratory distress. Her SpO_2_ had dropped dramatically to 69% on room air, and her respiratory rate increased to 33 cpm. She, like the first case, had no cough or fever, and there were still no abnormal findings on chest examination: no tenderness, abnormal percussion notes, crackles, or reduced air intensity throughout her admission. Her cardiovascular findings were also normal as of this time: normal pulse rates and BPs, and no heart murmurs.

#### 2.2.2 Differential Diagnosis, Investigations and Treatment

The differential diagnosis for her shortness of breath also included pneumonia, acute pulmonary edema from fluid overload, acute respiratory distress syndrome, and metabolic acidosis with respiratory compensation. Cholera was diagnosed by a stool culture following an RDT. Her complete blood count was unremarkable: hemoglobin (Hb)—11.3 g/dL, hematocrit—39%, white blood count (WBC)—7.5, and platelets—144. She had also been resuscitated with Ringer's lactate infusions and started on ORS within 4 h of admission. She was supported with intranasal oxygen at 4 L/min, which sustained her oxygen saturation above 95%, and received 300 mg of doxycycline as soon as her nausea improved (which was within 12 h of arrival).

#### 2.2.3 Outcome

By day 3 of admission, her stools were semi‐formed with reduced frequency, and she required less oxygen; the flow rate was reduced to 3 L/min. By day 4, she was discharged with complete resolution of symptoms.

### 2.3 Case 3

#### 2.3.1 Case History/Examination

This was a 5‐year‐old girl who presented with a 2‐day history of diarrhea and vomiting. She was severely dehydrated (slow skin recoil, altered mental status, sunken eyes, and delayed capillary refill). She came in with mild respiratory distress, which worsened shortly after admission: nasal flaring and tachypnea with Kussmaul breathing. Her SpO_2_ was persistently below 70% on room air; however, there were no abnormal chest findings on palpation, percussion, or auscultation.

#### 2.3.2 Differential Diagnosis, Investigations and Treatment

Like the first two cases, the differential diagnosis for her shortness of breath included pneumonia, acute respiratory distress syndrome, and metabolic acidosis with respiratory compensation. By protocol, cholera was diagnosed by RDT and confirmed by stool cultures. She also had an essentially normal CBC: Hb 12.0, WBC 7.9, platelets 434, hematocrit 41%. She received oxygen therapy, which kept her SpO_2_ above 95%, and was rehydrated with Ringer's lactate, cholera infusion 5:4:1 (sodium chloride: sodium bicarbonate: potassium chloride), and ORS, all calculated and administered according to her weight and degree of dehydration. She was also started on IV azithromycin and zinc tablets.

#### 2.3.3 Outcome

She remained in respiratory distress till day 3 of her admission but was afebrile throughout and finally discharged on day 4 of admission when all her symptoms had resolved.

## 3 Discussion

Though debilitating, respiratory complications of cholera are not well explored or documented. Recognized ones include pneumonia and acute pulmonary edema from fluid overload, followed by acute respiratory distress syndrome, which may be associated with respiratory failure. In addition to these, metabolic acidosis can result in Kussmaul breathing, which is physiologically a form of respiratory compensation for the metabolic acidosis [9]. There have been some reported cases of pulmonary cholera, pneumonia that developed after the individuals aspirated cholera‐contaminated freshwater; however, these were caused by non‐O1 *V. cholerae* [10].

Regarding pneumonia, vomiting in cholera patients increases the risk of aspiration, which can lead to aspiration pneumonitis and pneumonia. This is mostly seen in children [11]. Aspiration pneumonitis is a result of gastric acid causing a chemical pneumonitis, while aspiration pneumonia is purely infectious, with risks of pneumonia increasing with the volume of aspirated material [12, 13]. The absence of a fever, cough, or signs of consolidation on clinical examination of the listed cases, however, makes it improbable but possible, especially in the case of the child. This is because aspiration pneumonia can occur without significant chest findings, especially in the early stages [14]. The absence of radio imaging made it challenging to establish a definitive diagnosis, as the diagnostic process relied primarily on clinical examination.

Acute pulmonary edema from fluid overload results from aggressive intravenous rehydration. This condition can cause hypoxia without significant chest findings, as early interstitial edema may not manifest with overt signs. Pulmonary edema (cardiogenic) progresses in three stages, the first being vascular redistribution, followed by interstitial edema, and then alveolar edema [15]. Symptoms begin in the last 2 stages, but one may still be asymptomatic during interstitial edema on both chest exam and radiography. These patients, however, had unremarkable chest findings throughout admission. In addition, they received intravenous fluid in titrated quantities according to their level of dehydration and started on ORS immediately when they could drink to replace ongoing losses. These measures reduced the risks of fluid overload.

Acute respiratory distress syndrome (ARDS) is characterized by non‐cardiogenic pulmonary edema and hypoxia [16]. The disease course usually lasts 1–3 weeks [17]. Cholera may not cause ARDS directly but indirectly through extreme dehydration and hypovolemic shock, sepsis, electrolyte imbalances, and acute kidney injury, with sepsis being the commonest trigger [18]. Cholera is a gut‐localized infection; however, the severe dehydration and electrolyte imbalances it causes increase the susceptibility for other infections and sepsis through mechanisms like bacterial translocation, which allows for bacteria, including *V. cholerae* and other gut flora, to get into the bloodstream and other tissues [18]. During the outbreak in question, for instance, there were several cases that had a co‐infection with typhoid fever and had more severe symptoms and longer hospitalization. The patients in this case series had transient respiratory distress, with the longest duration being 72 h, which makes ARDS less likely.

Kussmaul breathing from metabolic acidosis is not typically associated with low oxygen saturations as was observed in these patients. It is characterized by long, deep breaths, which aim to expel more carbon dioxide to help compensate for metabolic acidosis [19]. It is not a diagnosis by itself, only a symptom. The cause of metabolic acidosis in cholera is multifactorial: loss of bicarbonate in stool, lactic acidosis from poor tissue perfusion, and impaired acid–base regulation in the case of acute kidney injury.

The discussed differentials for TARD in cholera do not completely explain the phenomenon that occurred in these patients. A differential not listed—pulmonary embolism—is another possibility that should be considered in all cases of acute respiratory distress. There is hemoconcentration that occurs in cholera resulting from severe dehydration if hydration therapy is not started early. This hemoconcentration causes venous stasis and increased coagulability, which, as part of Virchow's triad, can result in thrombi and hence emboli, which could cause pulmonary embolism. This has been well documented in primary and secondary polycythemia but not in hemoconcentration, though they are theoretically similar in terms of high hematocrit [20, 21]. The cases listed here all had normal hematocrit, which ruled out hemoconcentration.

**FIGURE 1:**
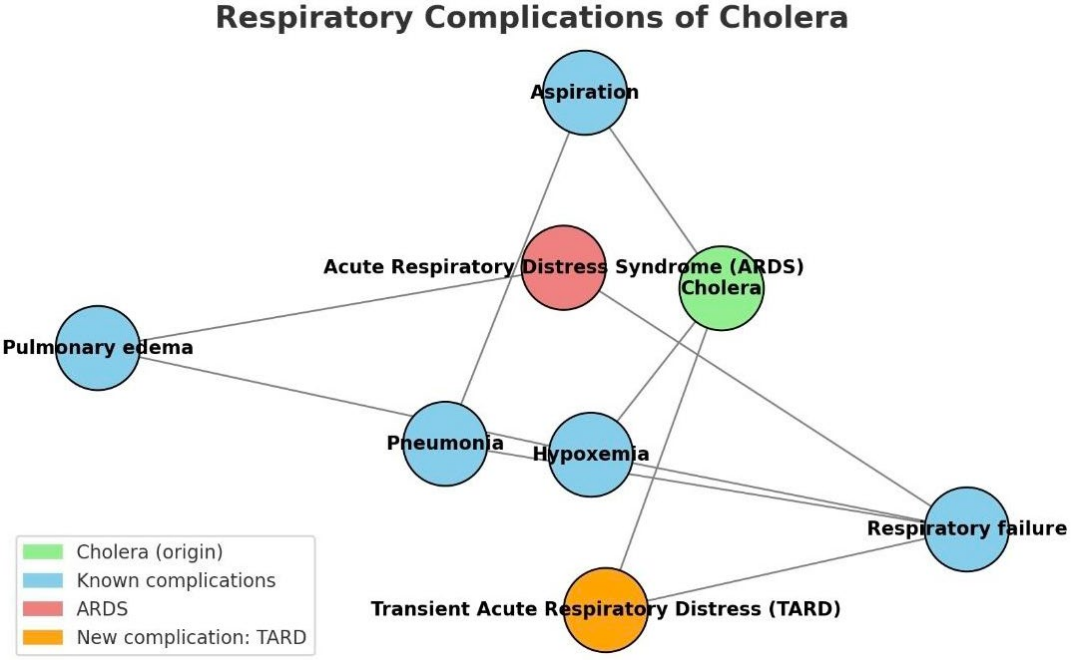
Network map illustrating the interconnections between cholera and its respiratory complications, including the newly observed transient acute respiratory distress (TARD).

TARD observed in these cholera patients, though inconclusive, likely resulted from a combination of dehydration, metabolic acidosis, fluid overload, and electrolyte imbalances. The absence of significant chest findings and the rapid resolution of symptoms suggest that these episodes were not due to primary pulmonary pathologies. The unavailability of radio imaging at the isolation bay also made the diagnosis more challenging. There may be another phenomenon that best explains TARD, but this requires further studies. Table 1 shows a summary of the case characteristics, and Figure 1 shows the interconnection between the respiratory complications of cholera.

**TABLE 1:** Characteristics of the three cholera patients who developed transient acute respiratory distress (TARD).

Parameter	Case 1	Case 2	Case 3
Onset of TARD after admission (h)	4	24	24
Duration of TARD (h)	4	36	72
Total time on admission (h)	24	76	80
Antibiotic received	Doxycycline	Doxycycline	Azithromycin
Sex	Male	Female	Female
Age (years)	23	37	5

## 4 Conclusion

Transient acute respiratory distress (TARD) in cholera patients is a multifactorial phenomenon that likely results from metabolic acidosis, fluid overload, and electrolyte imbalances. Awareness of this potential complication is crucial for clinicians managing cholera outbreaks. Further studies are needed to elucidate the underlying mechanisms and to develop targeted interventions to prevent and manage this condition.

## Author Contributions

**Samuel Amo‐Tachie:** conceptualization, data curation, formal analysis, investigation, methodology, visualization, writing – original draft, writing – review and editing.

## Consent

A written informed consent was obtained from the patients and guardians to publish this report in accordance with the journal's patient consent policy.

## Conflicts of Interest

The author declares no conflicts of interest.

## Data Availability

The data that support the findings of this study are available on request from the corresponding author [S.A.T.].
